# PfAP2-EXP2, an Essential Transcription Factor for the Intraerythrocytic Development of *Plasmodium falciparum*

**DOI:** 10.3389/fcell.2021.782293

**Published:** 2022-01-10

**Authors:** Xiaomin Shang, Changhong Wang, Li Shen, Fei Sheng, Xiaohui He, Fei Wang, Yanting Fan, Xiaoqin He, Mei Jiang

**Affiliations:** ^1^ Department of Medical Genetics, Shanghai Tenth People’s Hospital, School of Medicine, Tongji University, Shanghai, China; ^2^ Department of Parasitology, Xiangya School of Medicine, Central South University, Changsha, China; ^3^ National Health Commission Key Laboratory of Parasitic Disease Control and Prevention, Jiangsu Provincial Key Laboratory on Parasite and Vector Control Technology, Jiangsu Institute of Parasitic Diseases, Wuxi, China

**Keywords:** *Plasmodium falciparum*, ApiAP2 transcription factor PfAP2-EXP2, transcription regulation, asexual replication, cell remodeling

## Abstract

*Plasmodium falciparum* undergoes a series of asexual replications in human erythrocytes after infection, which are effective targets for combatting malaria. Here, we report roles of an ApiAP2 transcription factor PfAP2-EXP2 (PF3D7_0611200) in the intraerythrocytic developmental cycle of *P. falciparum*. PfAP2-EXP2 conditional knockdown resulted in an asexual growth defect but without an appreciable effect on parasite morphology. Further ChIP-seq analysis revealed that PfAP2-EXP2 targeted genes related to virulence and interaction between erythrocytes and parasites. Especially, PfAP2-EXP2 regulation of euchromatic genes does not depend on recognizing specific DNA sequences, while a CCCTAAACCC motif is found in its heterochromatic binding sites. Combined with transcriptome profiling, we suggest that PfAP2-EXP2 is participated in the intraerythrocytic development by affecting the expression of genes related to cell remodeling at the schizont stage. In summary, this study explores an ApiAP2 member plays an important role for the *P. falciparum* blood-stage replication, which suggests a new perspective for malaria elimination.

## Introduction

Malaria is still a major threat to public health with about two hundred million cases and four hundred thousand deaths each year (Li et al., 2019). *Plasmodium falciparum* infection begins when a mosquito bites a vertebrate host and injects sporozoites into its bloodstream. After the liver stage, parasites invade red blood cells and initiate repeated rounds of 48-h asexual replication cycle processing through ring, trophozoite and schizont stages. Parasites increase in number by multiplying in red blood cells and cause a series of symptoms like periodic fever and anemia (Josling and Llinás, 2015). In the blood stage, parasites could be sequestered and concentrated in tissues by adhesion to the host endothelium, leading to severe organ dysfunction (Wahlgren et al., 2017). Thus, it is the most useful strategy for malaria elimination to target the proliferative asexual life cycle (Haldar et al., 2018).

In *P. falciparum*, a total of 27 Apicomplexan AP2 (ApiAP2) family members have been identified, which is a conserved family known as containing at least one DNA binding domain similar to the Apetala2/ERF (ethylene response factor) (AP2/ERF) integrase domain in plants (Balaji et al., 2005; De Silva et al., 2008; Yamasaki et al., 2013; Jeninga et al., 2019). The majority of ApiAP2 transcription factors display stage-specific expression in the life cycle, suggesting that they might play distinct roles in the parasite development (Jeninga et al., 2019). So far, our knowledge about this family is limited to a few members. According to latest research, scientists have successfully knocked out 11 ApiAP2 transcription factors in *P. falciparum* and have gained a preliminary insight into their functions (Shang et al., 2021; Singh et al., 2021). PfAP2-G is recognized as the master positive regulator of sexual conversion between gametocyte commitment and development, while PfAP2-G5 and PfAP2-G2 work together as transcriptional repressors (Kafsack et al., 2014; Sinha et al., 2014; Poran et al., 2017; Xu et al., 2020; Shang et al., 2021; Singh et al., 2021). Another ApiAP2 family member, which is named PfAP2-I, has been reported to regulate the process of invasion (Santos et al., 2017). In addition, PfAP2-EXP is linked to transcriptional activation of the clonally variant exported protein families PfMC-2TM, RIFIN, and STEVOR (Martins et al., 2017). Periodic fever is a characteristic clinical feature of human malaria; PfAP2-HS regulates heat-shock response and protects human malaria parasites from febrile temperatures (Tintó-Font et al., 2021). In the asexual stage, PfSIP2 is involved in heterochromatin formation and genome integrity, and PfAP2-Tel is implicated in telomere biology (Flueck et al., 2010; Sierra-Miranda et al., 2017). There also exists an ApiAP2 factor, PfAP2-HC, without substantial function but as a core component of heterochromatin in malaria parasites (Carrington et al., 2021).

Here, we described the functions of an ApiAP2 factor, PF3D7_0611200, in the blood stage development of *P. falciparum* parasites by integrating multi-omics. We show that this transcription factor is required for the asexual development. Further functional investigations reveal that it is involved in the expression regulation of a number of genes encoding exported proteins. We thereby name this transcription factor as PfAP2-EXP2, since PfAP2-EXP has been identified previously (Martins et al., 2017).

## Materials and Methods

### Parasite Culture

*Plasmodium falciparum* 3D7-G7 strain was cultured in O type fresh human erythrocytes as described previously in complete RPMI 1640 medium (Gibco) with 0.5% Albumax I (Invitrogen) and a gas phase maintained under 5% CO_2_, 5%O_2_ and 90% N_2_ at 37°C (Zhang et al., 2014). Parasites were regularly synchronized with repeated 5% sorbitol treatments at the ring stage. For assays on *pfap2-exp2-ty1-glms* parasites, cultures were tightly synchronized to a 5-h window by purification of schizont stage using Percoll-sorbitol gradients (70% Percoll and 40% Percoll) followed by 5% sorbitol treatment 5 h later (Lu et al., 2021).

### Plasmid Construction

The *pL6cs-ap2-exp2-ty1-glms* and *pL6cs-ap2-exp2-ty1-GFP* plasmids were constructed as described previously (Ghorbal et al., 2014; Zhao et al., 2020). First, the guide RNA was annealed by complementary oligonucleotides and cloned into the *pL6cs* construct between *XhoI* and *AvrII* restriction enzyme sites. Then the C-terminal fragment of *pfap2-exp2* with ty1-glms or ty1-gfp was cloned into *AflII* and *AscI* restriction sites. The constructed plasmids verified by sequencing were transformed into *E. coli* XL10 for amplification and purification. All primers used for construction are listed in Supplementary Table S1.

### Generation of Transgenic Lines

Transfections were performed in uninfected red blood cells using 100 μg of purified *pL6cs-ap2-exp2-ty1-glms* or *pL6cs-ap2-exp2-ty1-GFP* plasmids together with 100 μg of pUF1-Cas9-BSD plasmids followed by the addition of purified schizont stage parasites (Zhao et al., 2020). Subsequently, parasites were cultured in the presence of 2.5 nM WR99210 and 2 μg/ml BSD (Invitrogen) until live parasites were refound in Giemsa’s solution-stained thin blood smears 3 weeks later. The sequences at the designed integration sites were examined by PCR of genomic DNAs followed by DNA sequencing to confirm genetic editing. The *pfap2-exp2-ty1-glms* strain was further cloned out by limiting dilution cloning (Fan et al., 2020). Primers used for verification are provided in Supplementary Table S1.

### Growth Curve Assays

*Pfap2-exp2-ty1-glms* parasites were tightly synchronized to a 5-h window. Ring-stage parasites were plated at 0.5% parasitemia in a 6-well plate with 2% hematocrit in the presence or absence of 5 mM glucosamine (GlcN). Parasitemia was monitored periodically by counting parasites from Giemsa-stained thin blood smears in the next two cycles (Liu et al., 2020).

### Western Blots

Sample preparation for western blot analysis was performed as previously described (Liu et al., 2020). Briefly, schizonts were released from erythrocytes with 0.15% saponin, resuspended with an equal volume of 2 × SDS–polyacrylamide gel electrophoresis (PAGE) protein loading buffer, and then heated for 5 min at 100°C before storing at -80°C. Proteins were separated by 10% SDS-PAGE, and then transferred to Immobilon-P transfer membranes (Millipore). Subsequent antibody incubation and membrane wash followed standard procedures. The primary antibodies used in this study included mouse anti-ty1 (Sigma, SAB4800032) at 1:1,000 and rabbit anti-aldolase (Abcam, ab207494) at 1:2,000. The horseradish peroxidase (HRP) conjugated secondary antibodies were used at 1:5,000, including goat anti-mouse IgG (Abcam, ab97040) and goat anti-rabbit IgG (Abcam, ab205718). HRP signals were detected using the ECL western blotting kit (GE healthcare). Especially, *pfap2-exp2-ty1-glms* parasites were tightly synchronized to a 5-h window and ring-stage parasites were diluted at 0.5% parasitemia with 2% hematocrit in the presence or absence of 5 mM glucosamine. At the second generation, 200 μL of schizont-staged samples were collected for western blotting.

### Immunofluorescence Assays

The immunofluorescence assay was performed to detect the localization of PfAP2-EXP2 as described previously (Jing et al., 2018). Parasites were harvested, released, fixed by 4% paraformaldehyde (Electron Microscopy Sciences) at room temperature for 10 min, and washed by PBS. Prepared samples were incubated with the primary antibodies against ty1 (Sigma, SAB4800032) or GFP (Abcam, ab290) at 1:500 to 1:1,000, followed by the secondary antibodies AlexaFluor 488 goat anti-mouse IgG (ThermoFisher Scientific, A11029) or AlexaFluor 568 goat anti-rabbit IgG (ThermoFisher Scientific, A11036) at 1:500. Preparations were visualized with a Nikon A1R microscope at 60-100 × magnification and images were acquired with NIS Elements software and processed using Adobe Photoshop.

### RNA-Seq

*Pfap2-exp2-ty1-glms* parasites were tightly synchronized to a 5-h window with or without 5 mM glucosamine treatment as described above. Samples were collected in TRIzol at ring (10–15 hpi, one biological replicate), trophozoite (25–30 hpi, two biological replicates) and schizont (40–45 hpi, three biological replicates) stages of the next cycle, respectively. Total RNA was extracted according to the manufacturer’s protocol (Zymo Research). Library preparation for strand-specific RNA-seq was first carried out by poly(A) selection with the KAPA mRNA Capture Beads (KAPA) and fragmentation to about 300–400 nucleotides (nt) in length. All subsequent steps were performed according to the KAPA Stranded mRNA-Seq Kit (KK8421). Libraries were sequenced on an Illumina HiSeq Xten system to generate 150 bp pair-end reads (Liu et al., 2019; Lu et al., 2021).

### Chromatin Immunoprecipitation Sequencing

ChIP-seq assays were carried out in two biological replicates as previously described with minor modifications (Lopez-Rubio et al., 2013; Santos et al., 2017; Josling et al., 2020). Synchronized parasites were harvested at schizont stage and cross-linked immediately with 1% paraformaldehyde (Sigma) by rotating for 10 min at 37°C, then quenched with 0.125 M glycine for 5 min on ice. The parasites were resuspended with 50 ml of PBS and lysed with 0.15% saponin for 5 min on ice. The released nuclei were washed several times with PBS, then sonicated using an M220 sonicator (Covaris) at 5% duty factor, 200 cycles per burst, and 75 W of peak incident power to generate 100–500 bp fragments. The samples were diluted ten-fold with dilution buffer and precleared with Protein A/G magnetic beads (Thermo) for 2 hours at 4°C. The precleared chromatin supernatants, a small part of which were aliquoted as input controls, were incubated overnight at 4°C with 0.5 μg of antibodies against GFP (Abcam, ab290) and 20 μL of Protein A/G magnetic beads. The immunoprecipitates were washed with low salt wash buffer, high salt wash buffer, LiCl wash buffer, and TE buffer, then eluted with Elution Buffer. To reverse cross-link, the eluted samples were incubated overnight at 45°C and treated with RNase A at 37°C for 30 min and Proteinase K at 45°C for 2 hours. Finally, DNA was extracted using the MinElute PCR purification kit (Qiagen, 28006). In library preparation, 1.5 ng of ChIP-DNAs were end-repaired (Epicentre No. ER81050), added with protruding 3’ A base (NEB No. M0212L), and ligated with adapters (NEB No. M2200L). The Agencourt AMPure XP beads (Beckman Coulter) were then used for size selection and purification. Libraries were amplified using the KAPA HiFi PCR Kit (KAPA Biosystems, KB2500) with the following program: 1 min at 98°C; 12 cycles of 10 s at 98°C and 1 min at 65°C; finally, extension 5 min at 65°C. Library sequencing was conducted on an Illumina HiSeq Xten platform and generated 150 bp pair-end reads.

### ChIP-Seq Analysis

To remove residual adapters and low-quality bases, read trimming was conducted with Trimmomatic (Bolger et al., 2014) using a 4 bp window and average window quality above 15. Clipped reads with a minimum length of 50 bp and average read quality above 20 were mapped to the *P. falciparum* 3D7 genome build 47 using Bowtie2 (Langmead and Salzberg, 2012) and default parameters. Peaks were identified using the--call-summits option of the MACS2 callpeak function (Zhang et al., 2008) and a q-value cutoff of 0.05. Log2-transformed ChIP/input fold enrichment signals were calculated with the MACS2 bdgcmp function and visualized with Gviz (Hahne and Ivanek, 2016).

GenomicRanges (Lawrence et al., 2013) assigned peaks to nearby target genes if they overlapped 5′ UTRs (<3 kb upstream of the translation start sites), gene body, and 3’ UTRs (<0.5 kb downstream of the translation stop sites). Functions enriched in the target genes were analyzed using malaria parasite metabolic pathways (MPMP) (Ginsburg, 2006), functional gene families, and clusterProfiler (Yu et al., 2012) [Benjamini-Hochberg (BH) adjusted *p*-value of <0.01].

Peaks were extended +/− 250 bp around summits. Those detected in both biological replicates were reserved for discovery of PfAP2-EXP2 DNA binding motifs between 6 bp and 10 bp using DREME (Bailey, 2011) as compared with random genomic regions of 500 bp.

### RNA-Seq Analysis

RNA-seq reads were trimmed as described in the ChIP-seq analysis, then aligned to the 3D7 genome using HISAT2 (Kim et al., 2015) with the guide by the gene annotation and default parameters except--max-intronlen 5,000 --dta--rna-strandness RF. StringTie (Pertea et al., 2016) counted reads mapped to genes. Subsequently, edgeR (Robinson et al., 2010) analyzed differential gene expression (fold change of >2 and false discovery rate of <0.05). Over-representation analyses of MPMP pathways were performed on the differentially expressed genes using clusterProfiler (BH adjusted *p*-value of <0.01).

## Results

### PfAP2-EXP2 is Required for the Blood-Stage Development of *P. falciparum*

**FIGURE 1:**
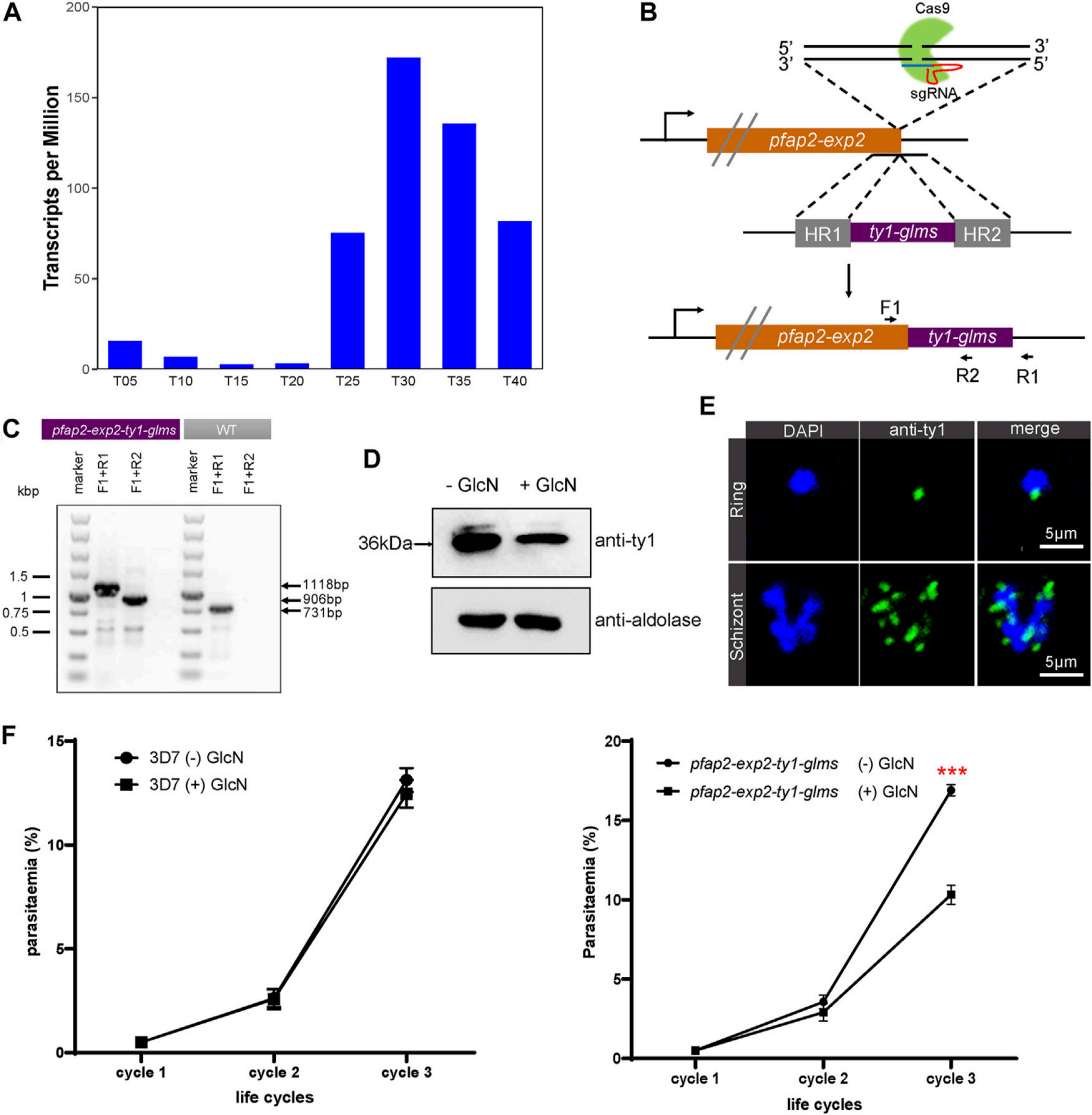
PfAP2-EXP2 transcription factor is required for the blood-stage parasite development. **(A)** Expression profiles of *pfap2-exp2* in *Plasmodium falciparum* 3D7 strain throughout the intraerythrocytic developmental cycle according to RNA-seq transcriptomic analysis (Toenhake et al., 2018). **(B)** Incorporation of a *pfap2-exp2-ty1-glms* construct into the *pfap2-exp2* locus of the wildtype 3D7-G7 line by homologous recombination. F1, R1, and R2 indicate locations of diagnostic PCR primers. **(C)** The glucosamine inducible knockdown transgenic parasite line was confirmed by diagnostic PCR using primers indicated in **(B)**. **(D)** Western blotting proved effective knockdown of the *pfap2-exp2* gene by the glucosamine (GlcN) addition in the *3D7/pfap2-exp2-ty1-glms* transgenic parasite line. **(E)** Immunofluorescence assays using anti-ty1 revealed perinuclear distribution of PfAP2-EXP2 in the 3*D7/pfap2-exp2-ty1-glms* rings and schizonts. **(F)** Growth curves of 3D7-G7 (left panel) and the *3D7/pfap2-exp2-ty1-glms* line (right panel) in the presence or absence of glucosamine (+/− GlcN). Comparisons between (+) GlcN and (−) GlcN treatments were performed with student’s *t*-tests. *** indicates *p*-value < 0.001.

Based on time-series expression profiling of intraerythrocytic developmental cycle in *Plasmodium falciparum* 3D7 strain (Toenhake et al., 2018), the mRNA level of *pfap2-exp2* reaches its peak at trophozoite and schizont stages (Figure 1A). To gain an insight into the function of PfAP2-EXP2, we first attempted to directly disrupt its gene using the CRISPR/Cas9 knockout system, but it failed after five independent transfections with two different sgRNAs (data not shown). We speculated that PfAP2-EXP2 might be essential for parasite development during the asexual replications. Afterwards, a conditional gene knockdown strategy was adopted, which introduced a glucosamine inducible *glms* riboswitch element into the 3’ end of *pfap2-exp2* gene (Figure 1B) (Liu et al., 2020; Carrington et al., 2021). After drug selection and limiting dilution cloning, we successfully obtained a 3D7/*pfap2-exp2-ty1-glms* transgenic knockdown parasite line. Then the genomic DNA of the transgenic parasite line was collected, and two sets of primers were used for conventional PCR detection to verify the correct editing of the *pfap2-exp2* locus (Figure 1C). Furthermore, to examine knockdown efficiency, tightly synchronized ring stage 3D7/*pfap2-exp2-ty1-glms* parasites within 5 h window were treated with or without 5 mM glucosamine (GlcN) and harvested at late trophozoite and early schizont stages of the next generation. PfAP2-EXP2 protein level dramatically decreased upon exposure to the glucosamine (Figure 1D), indicating effective knockdown of the *pfap2-exp2* gene by the *glms* riboswitch system.

PfAP2-EXP2 was detected at the nuclear periphery in both rings and schizonts (Figure 1E), suggesting its involvement in transcriptional regulation the same as other ApiAP2 members (Josling et al., 2020; Singh et al., 2021). To investigate the effect of PfAP2-EXP2 on parasite viability, the conditional knockdown parasite line was tightly synchronized to a 5-h window and maintained with or without glucosamine. Repeated monitoring was performed by Giemsa-stained thin blood smears over three consecutive generations. Parental strain 3D7-G7 was also included in the growth curve analysis as a wild type control (Liu et al., 2020). Partial loss of PfAP2-EXP2 led to an approximate 40% reduction in the parasite growth rate (student’s *t*-test *p*-value < 0.001, Figure 1F). We examined the thin blood smears of the last cycle and found that the *pfap2-exp2-ty1-glms* parasites treated by the glucosamine still retained normal morphology. In conclusion, PfAP2-EXP2 plays an important role in the asexual proliferation of *P. falciparum*, but does not affect its morphology.

### Role of PfAP2-EXP2 in Transcriptional Regulation at the Schizont Stage

**FIGURE 2:**
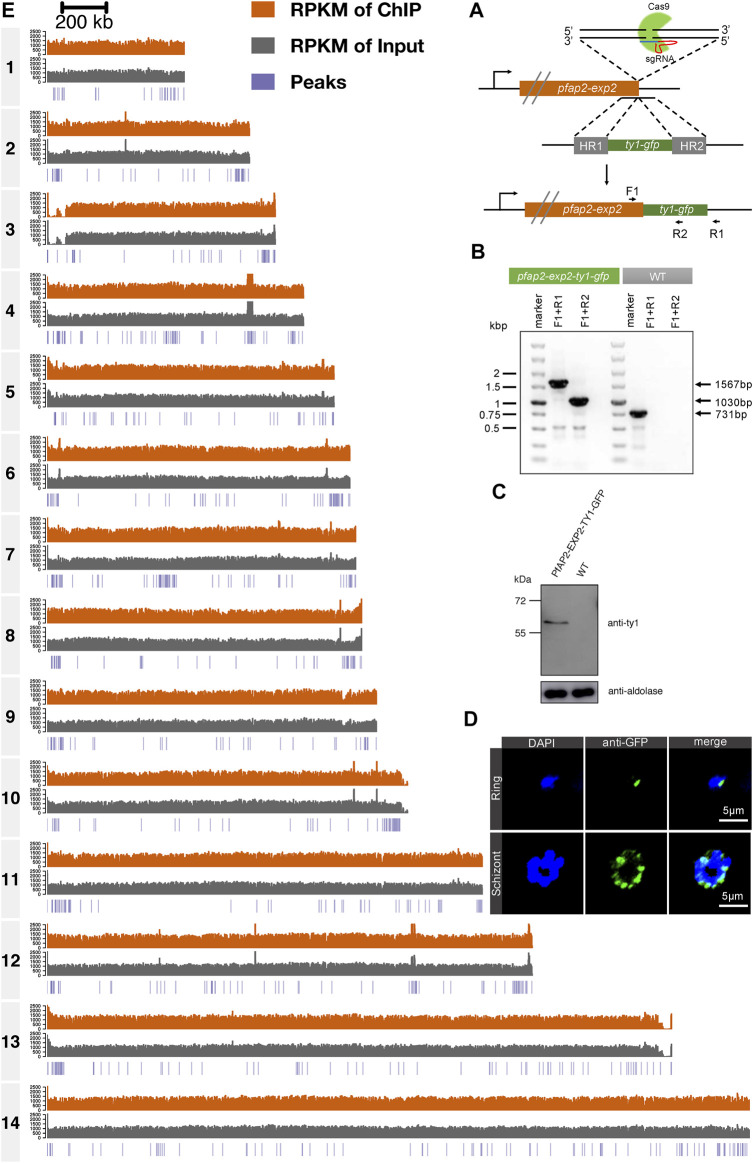
Genome-wide occupancies of PfAP2-EXP2 at the schizont stage. **(A)** Generation of a *pfap2-exp2* transgenic parasite line tagged with GFP at the C-terminus using the CRISPR/Cas9 gene editing system. **(B)** The GFP tagged PfAP2-EXP2 transgenic line was verified by diagnostic PCR using F1, R1, and R2 primers. **(C)** The expression of PfAP2-EXP2 in the *3D7/pfap2-exp2-ty1-gfp* transgenic line was validated by western blotting. **(D)** Immunofluorescence assays using anti-GFP demonstrated perinuclear distribution of PfAP2-EXP2 in the *3D7/pfap2-exp2-ty1-gfp* rings and schizonts. **(E)** Reads per kilobase of sequence range per Million mapped reads (RPKM) of ChIP, RPKM of input, and peaks in chromosomes 1 to 14 at the schizont stage detected by one of the two biological replicates of ChIP-seq for PfAP2-EXP2.

Given the perinuclear distribution of PfAP2-EXP2, ChIP-seq assays were performed to explore its role in transcriptional regulation. To achieve this, we first constructed a C-terminal GFP tagged PfAP2-EXP2 transgenic parasite line using the CRISPR/Cas9 gene editing system (Figure 2A). The newly developed *pfap2-exp2-ty1-gfp* strain was verified by diagnostic PCR (Figure 2B). Western blotting validated the expression of PfAP2-EXP2 in this transgenic parasite line (Figure 2C). Moreover, immunofluorescence assays confirmed the perinuclear localization of PfAP2-EXP2 in rings and schizonts (Figure 2D).

**FIGURE 3:**
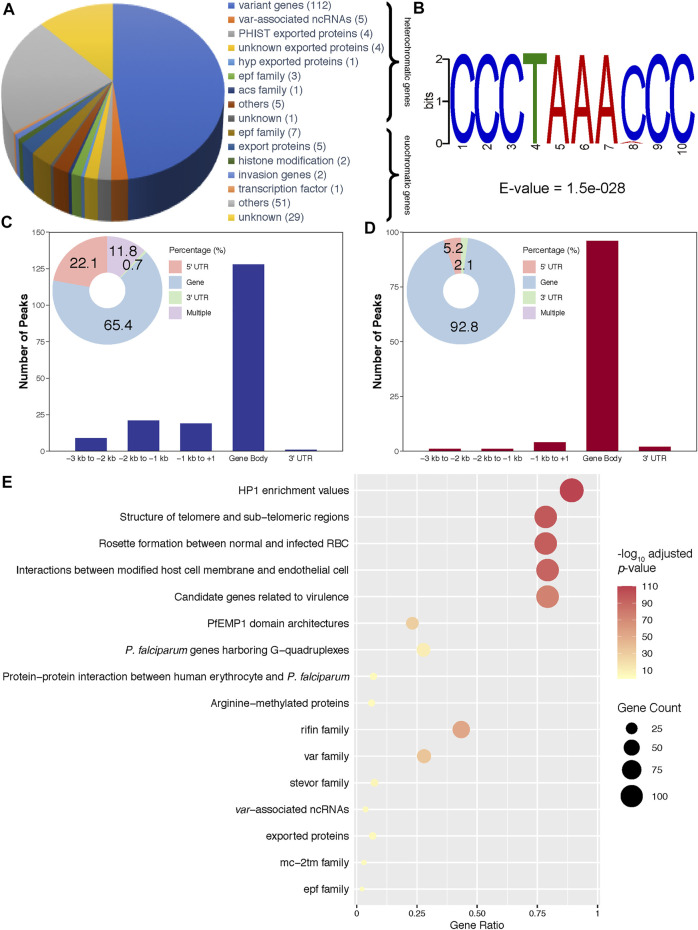
Characteristics of PfAP2-EXP2 occupancy. **(A)** Constitution of PfAP2-EXP2 target genes. Gene numbers are listed after functional categories. **(B)** A DNA motif discovered in heterochromatic binding sites. **(C)** PfAP2-EXP2 binding distribution at heterochromatic gene loci. **(D)** PfAP2-EXP2 binding distribution at euchromatic gene loci. **(E)** MPMP pathways and functional families enriched in PfAP2-EXP2 heterochromatic target genes at the schizont stage (BH adjusted enrichment *p*-values of <0.01).

Then, to investigate transcriptional regulation by PfAP2-EXP2, ChIP-seq assays were performed on the *pfap2-exp2-ty1-gfp* schizonts using antibodies against GFP, which showed good reproducibility with a correlation higher than 0.9 (Pearson correlation coefficient = 0.96, Figure 2E and Supplementary Figure S1). PfAP2-EXP2 binding sites were observed near a total of 233 genes, including 136 heterochromatic genes and 97 euchromatic genes (Figure 3A; Supplementary Table S2), which suggested a strong preference of this ApiAP2 factor for targeting heterochromatic genes (BH adjusted hypergeometric test *p*-value = 3.09e-81 and enrichment ratio of heterochromatin targeting to euchromatin targeting = 17). In addition, no motifs were detected in euchromatic PfAP2-EXP2 binding sites, which is consistent with the previous report on motif characterization using protein binding microarrays (Campbell et al., 2010). Intriguingly, a specific DNA motif CCCTAAACCC was found at the heterochromatic PfAP2-EXP2 binding sites (Figure 3B). Thus, PfAP2-EXP2 might regulate heterochromatic and euchromatic genes by different mechanisms. PfAP2-EXP2 is mostly bound to the gene body regions of both heterochromatic and euchromatic genes (Figures 3C,D). A notable part of occupancies was also observed at 5’ UTR regions. Malaria Parasite Metabolic Pathway (MPMP) and functional family analyses of heterochromatic target genes revealed that PfAP2-EXP2 might participate in regulating multiple crucial pathways for parasite growth and development, such as HP1 enrichment values, structure of telomere and sub-telomeric regions, rosette formation between normal and infected RBCs, interactions between modified host cell membrane and endothelial cell, and candidate genes related to virulence (Figure 3E; Supplementary Table S3). No MPMP pathways were found to be enriched in PfAP2-EXP2 euchromatic target genes.

### PfAP2-EXP2 Impacts Gene Transcription Mainly at the Schizont Stage

**FIGURE 4:**
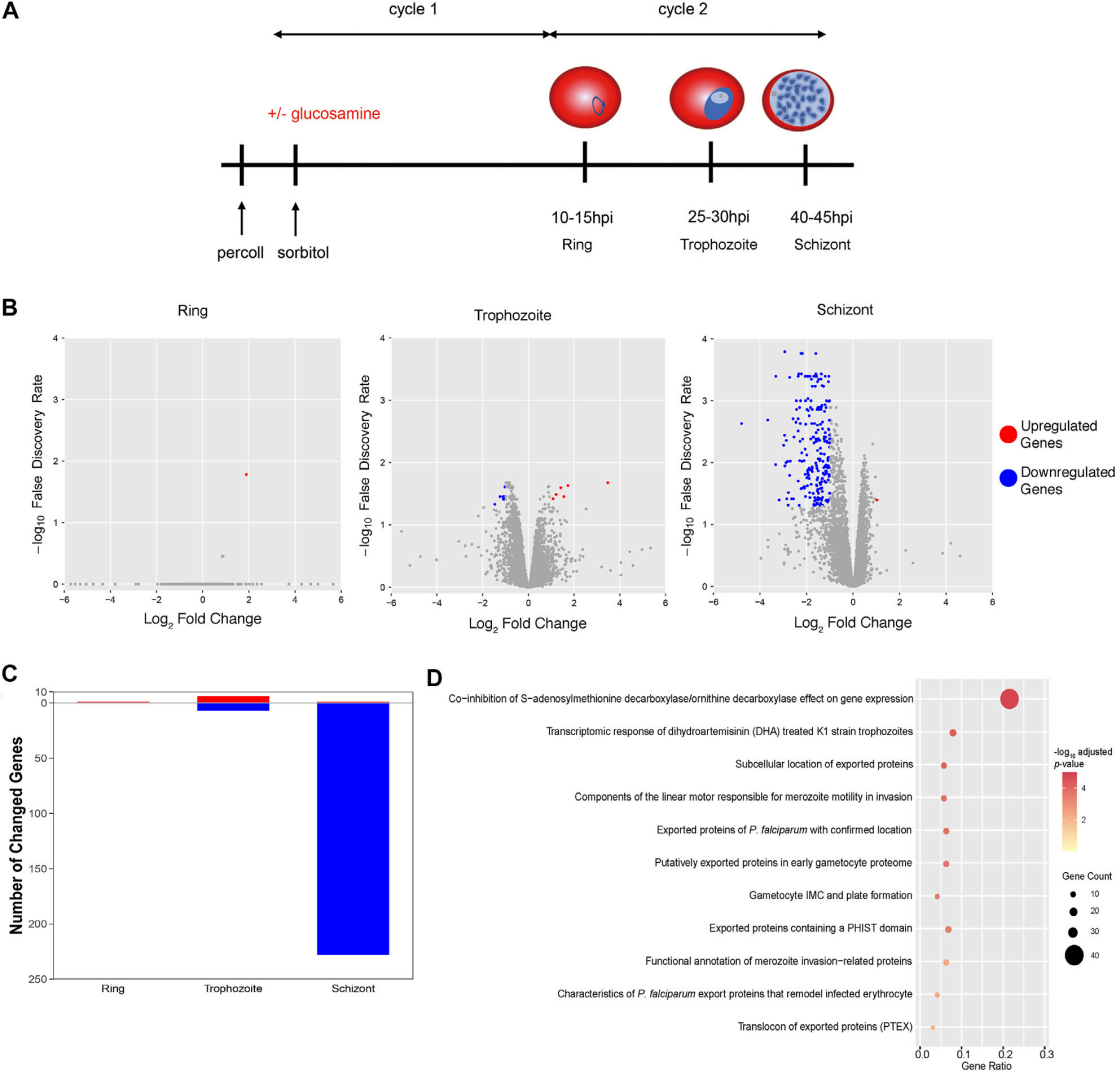
Impacts of PfAP2-EXP2 on gene transcription. **(A)** The scheme of parasite collection for RNA-seq assays. **(B)** Transcriptome changes in the PfAP2-EXP2 knockdown rings, trophozoites, and schizonts, respectively (fold change of >2 and false discovery rate of <0.05). **(C)** Numbers of genes differentially expressed in the PfAP2-EXP2 knockdown rings, trophozoites, and schizonts, respectively. **(D)** MPMP pathways enriched in genes with expression downregulated by the PfAP2-EXP2 knockdown at the schizont stage (BH adjusted enrichment *p*-values of <0.01).

To inspect roles of PfAP2-EXP2 in the asexual cycle of *P. falciparum*, we performed transcriptome analyses on the *pfap2-exp2-ty1-glms* parasites. Parasites were tightly synchronized to a 5-h window and cultured in the presence or absence of glucosamine (Figure 4A). After reinvasion, they were collected for RNA-seq at three time points, including 10–15 hpi, 25–30 hpi and 40–45 hpi (Figure 4A). The global transcriptome did not change much in the rings and trophozoites after PfAP2-EXP2 knockdown, and only less than 15 genes showed expression changes (Figures 4B,C; Supplementary Tables S4, S5). However, appreciable effects of PfAP2-EXP2 on gene transcription were observed at the schizont stage (Figures 4B,C; Supplementary Table S6). A total of 229 genes were altered on the expression level, out of which, 228 genes were downregulated by more than two times. Therefore, PfAP2-EXP2 regulates gene transcription mainly at the schizont stage, which is consistent with its peak expression from mid to late schizont stage. Furthermore, the genes downregulated in the PfAP2-EXP2 knockdown line included genes coding for exported proteins, invasion genes and genes involved in transcriptomic response of DHA treated K1 strain trophozoites (Figure 4D; Supplementary Table S7; Supplementary Figure S2), the first two functional groups of which are especially essential for parasite growth (Cowman et al., 2017; Frénal et al., 2017; Warncke and Beck, 2019).

### PfAP2-EXP2 Probably Participates in Cell Remodeling

**FIGURE 5:**
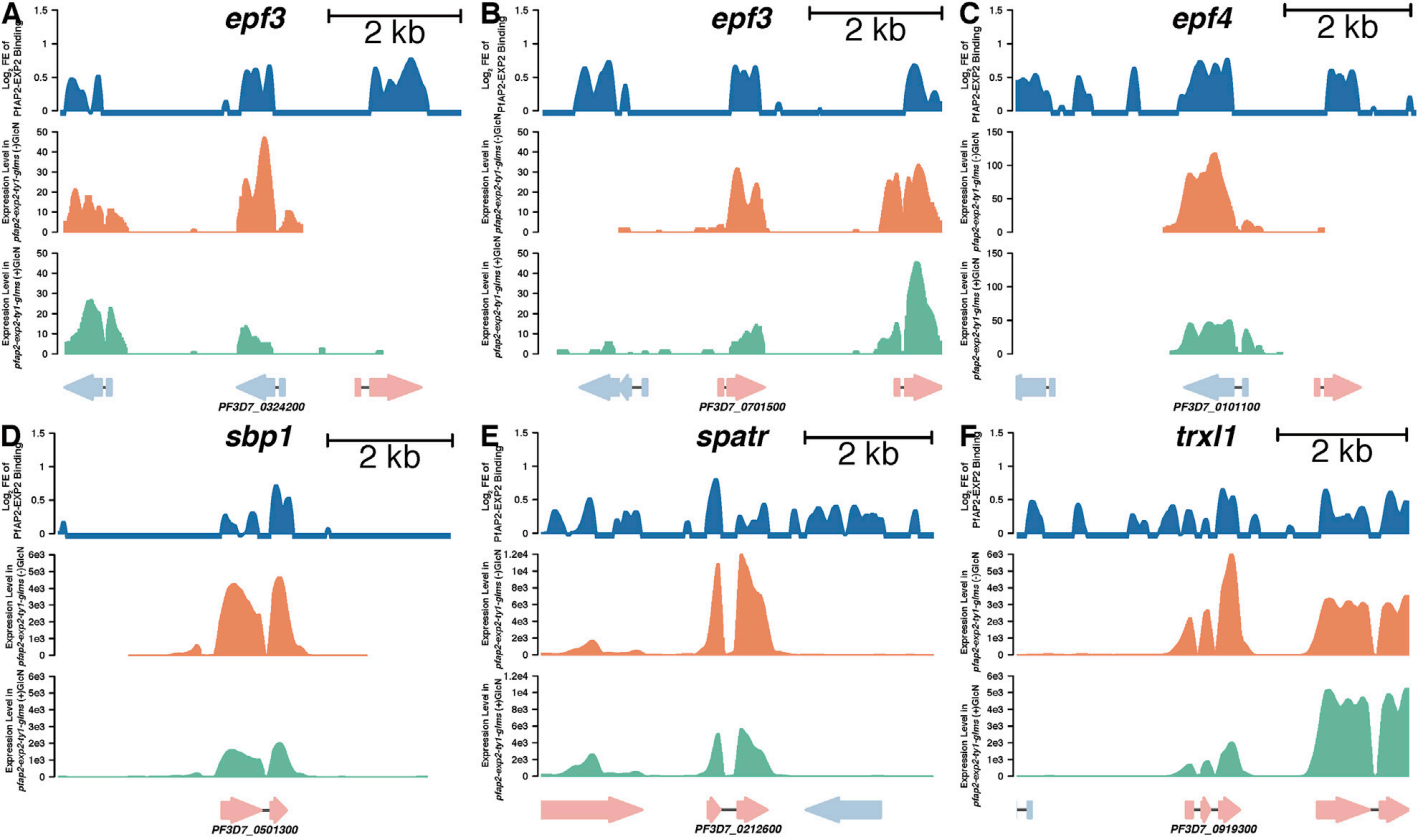
PfAP2-EXP2 binding sites at representative genes and their expression changes caused by the PfAP2-EXP2 knockdown at the schizont stage, including *epf3* **(A, B)**, *epf4* **(C)**, *sbp1* **(D)**, *spatr* **(E)**, and *trxl1* **(F)**. For each gene locus, from the top to the bottom panels: log_2_ fold enrichment (FE) of PfAP2-EXP2 binding, reads per kilobase per million mapped fragments (RPKM) in the 3D7/*pfap2-exp2-ty1-glms* line without glucosamine treatment, and RPKM in the 3D7/*pfap2-exp2-ty1-glms* line with glucosamine treatment.

Given that knockdown of *pfap2-exp2* led to defects in the growth of asexual cycles, we focused on its impacts on exported proteins that are critical for cell remodeling. Ten genes coding for exported proteins were directly targeted by PfAP2-EXP2 with more than 50% expression reduction by the PfAP2-EXP2 knockdown as well, including EPF3 (Figures 5A,B), EPF4 (Figure 5C), and variant gene clusters. In addition, PfAP2-EXP2 directly activated skeleton-binding protein 1 (SBP1) (Figure 5D), secreted protein with altered thrombospondin repeat domain (SPATR) (Figure 5E), and thioredoxin-like protein 1 (TrxL1) (Figure 5F). SPATR protein is expressed around the rhoptries at the asexual erythrocytic stage and related to merozoite invasion (Chattopadhyay et al., 2003; Huynh et al., 2014). SBP1 plays an important role in transporting molecules to the surface of infected erythrocytes (Kats et al., 2015; Iriko et al., 2020). Even though the exact function of TrxL1 in *Plasmodium falciparum* is poorly understood, its orthologue in *Toxoplasma gondii* is a subunit of a microtubule-associated complex which regulates the cellular cytoskeleton in the cells (Liu et al., 2013). Thus, we speculated that PfAP2-EXP2 might affect the growth of *Plasmodium falciparum* in the asexual cycle through regulating cell remodeling process.

## Discussion

As the largest transcription factor family in *Plasmodium*, some ApiAP2 members are believed to be critical for the parasite development, such as AP2-I and AP2-G (Kafsack et al., 2014; Sinha et al., 2014; Santos et al., 2017). The function of *pfap2-exp2* is still unclear. It can be mutated and is predicted to be nonessential for intraerythrocytic developmental cycle according to large-scale mutagenesis studies (Bushell et al., 2017; Zhang et al., 2018). However, we failed in knockouting *pfap2-exp2* with attempts to delete a part of its CDS. Furthermore, our present study showed that PfAP2-EXP2 conditional knockdown led to growth restriction suggesting the requirement of PfAP2-EXP2 for the intraerythrocytic developmental cycle of *P. falciparum*, which is consistent with the role of its orthologue in *P. yoelii* (Zhang et al., 2017).

Based on perinuclear distribution, genome occupancy, and effects on gene expression, PfAP2-EXP2 functions as a transcriptional activator, which is stage-specific mainly at the schizont stage. In addition, its transcriptional regulation is highly restricted to genes participating in cell remodeling. In order to adapt to the host environment, malaria parasites excrete many proteins to the erythrocyte surface to alter the permeability and adhesion of the erythrocytes. The cell remodeling process aids in intracellular iron homeostasis and nutrient uptake to maintain parasite development (Yam et al., 2017; Beck and Ho, 2021; Jonsdottir et al., 2021). The most common exported proteins include knob associated histidine rich protein (KAHRP), ring-infected erythrocyte surface antigen (RESA), the *Plasmodium* helical interspersed subtelomeric-domain proteins (PHIST), and exported protein family (EPF) (Warncke et al., 2016; Matthews et al., 2019; Warncke and Beck, 2019). PfAP2-EXP2 can directly activate transcription of a set of cell remodeling-related genes, such as *epf3, epf4, spatr, sbp1*, and *trxl1*. Although the specific roles played by these proteins in the cell remodeling process are still unclear, we believe that they are associated with the impact of PfAP2-EXP2 on the parasite growth. In addition, PfAP2-EXP2 is probably engaged in artemisinin response. Artemisinin resistance is emerging as a new challenge to the globe despite more than a century of efforts to control and eradicate malaria (Haldar et al., 2018).

Interestingly, PfAP2-EXP2 shows a preference for targeting heterochromatic genes. Furthermore, PfAP2-EXP2 displays distinct patterns of regulating euchromatic and heterochromatic genes. Its recognition of euchromatic genes does not depend on specific DNA sequences.

Collectively, we characterized an ApiAP2 transcription factor PfAP2-EXP2 that plays an important role in the asexual replication cycle of *P. falciparum*. We propose that one of its functions is regulating the expression of genes coding for cell remodeling proteins which are closely related to the physiology and pathology of *P. falciparum*, and other metabolic mechanisms it is involved in need to be further investigated. The present study enables us to rethink the role of ApiAP2 transcription factor in transcriptional regulation and also sheds a new light on the investigation of the metabolic mechanism of *P. falciparum*.

## Data Availability

The datasets presented in this study can be found in online repositories. The names of the repository/repositories and accession number(s) can be found below: https://www.ncbi.nlm.nih.gov/geo/, GSE180438.
