# Supplementary figures and images for: PfAP2-EXP2, an Essential Transcription Factor for the Intraerythrocytic Development of Plasmodium falciparum
Source: Front Cell Dev Biol. 2022 Jan 10;9:782293. doi: 10.3389/fcell.2021.782293 (PMC8785209; doi:10.3389/fcell.2021.782293)

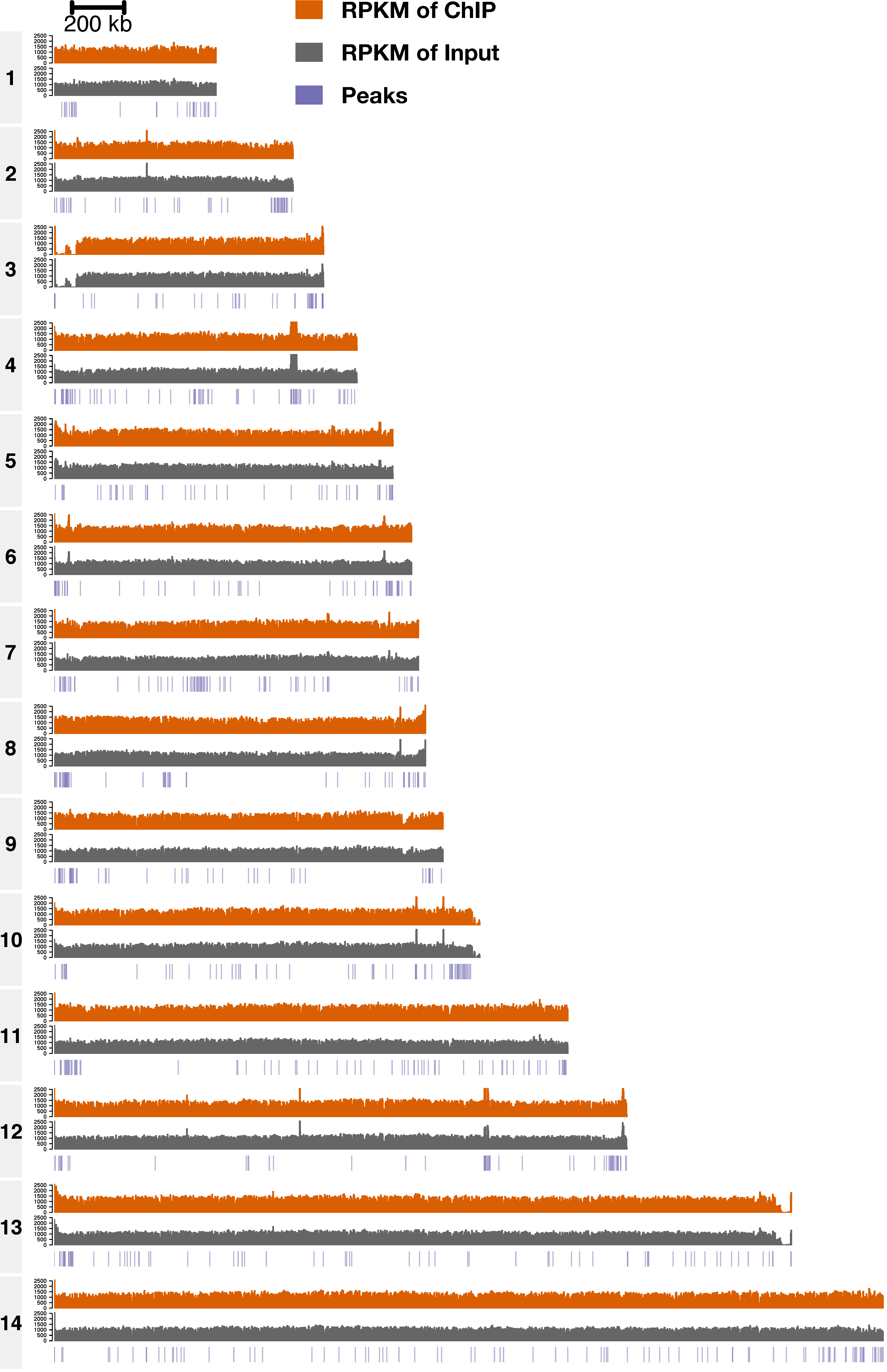

Supplement: Supplementary file 2 [file Image1.TIFF]

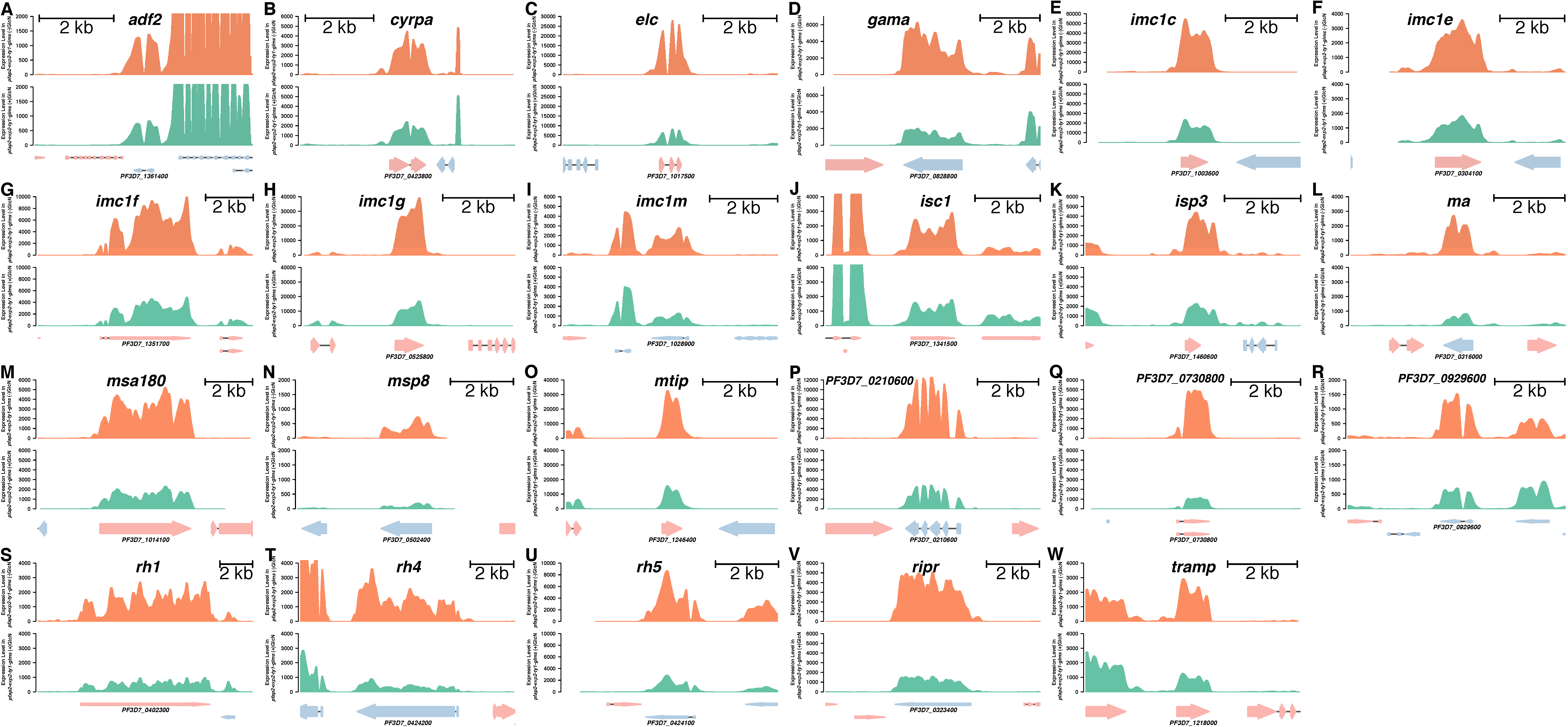

Supplement: Supplementary file 8 [file Image2.TIFF]
